# Motor and Cognitive Performance in Patients with Liver Cirrhosis with Minimal Hepatic Encephalopathy

**DOI:** 10.3390/jcm9072154

**Published:** 2020-07-08

**Authors:** Constanza San Martín-Valenzuela, Aroa Borras-Barrachina, Juan-José Gallego, Amparo Urios, Víctor Mestre-Salvador, Patricia Correa-Ghisays, María-Pilar Ballester, Desamparados Escudero-García, Joan Tosca, Cristina Montón, María-Pilar Ríos, Elena Kosenko, Vicente Felipo, Rafael Tabares-Seisdedos, Gabriel Selva-Vera, Carmina Montoliu

**Affiliations:** 1 Unit of Personal Autonomy, Dependency and Mental Disorder Assessment, Faculty of Medicine, University of Valencia, 46010 Valencia, Spain; constanza.martin@uv.es (C.S.M.-V.); borrasba@alumni.uv.es (A.B.-B.); victor.mestre@uv.es (V.M.-S.); rafael.tabares@uv.es (R.T.-S.); 2 Department of Physiotherapy, Faculty of Physiotherapy, University of Valencia, 46010 Valencia, Spain; 3 Centro Investigación Biomédica en Red de Salud Mental, CIBERSAM, 28029 Madrid, Spain; patricia.correa@uv.es; 4 INCLIVA, Health Research Institute, 46010 Valencia, Spain; jjgallego@incliva.es (J.-J.G.); aurios@incliva.es (A.U.); mapibafe@gmail.com (M.-P.B.); 5 Faculty of Psychology, University of Valencia, 46010 Valencia, Spain; 6 Digestive Medicine Unit, Hospital Clinico Universitario de Valencia, 46010 Valencia, Spain; m.desamparados.escudero@uv.es (D.E.-G.); joantosca@gmail.com (J.T.); Cris_monton@hotmail.com (C.M.); 7 Department of Medicine, Faculty of Medicine, University of Valencia, 46010 Valencia, Spain; 8 Digestive Service, Arnau de Vilanova Hospital, 46015 Valencia, Spain; mriosp73@hotmail.com; 9 Institute of Theoretical and Experimental Biophysics of Russian Academy of Sciences, 142290 Pushchino, Russia; eakos@rambler.ru; 10 Laboratory of Neurobiology, Príncipe Felipe Research Center, 46012 Valencia, Spain; vfelipo@cipf.es; 11 Unit of Psychiatry and Psychological Medicine, Hospital Clinico Universitario de Valencia, 46010 Valencia, Spain; 12 Department of Pathology, Faculty of Medicine, University of Valencia, 46010 Valencia, Spain

**Keywords:** minimal hepatic encephalopathy, biomechanics measurements, cognitive assessment

## Abstract

Minimal hepatic encephalopathy (MHE) is associated with mild cognitive impairment and frailty. This study aims to identify cognitive and motor differences in cirrhotic patients with and without MHE, and the correlations between motor signs and cognitive performance. Gait, balance, hand strength and motor speed performance were evaluated in 66 cirrhotic patients (38 without and 28 with MHE, according to the Psychometric Hepatic Encephalopathy Score (PHES). Cognitive performance was measured with the Mini-Mental State Examination, Verbal Fluency Test, Aprendizaje Verbal España-Complutense Test (TAVEC), Wechsler Adult Intelligence Scale III, Hamilton Depression and Anxiety Rating Scale and Functioning Assessment Short Test (FAST). MHE patients performed worse than patients without MHE in cognitive and autonomous functioning, learning and long-term memory, and verbal fluency. The same pattern was found in gait, center of pressure movement, variability of hand strength performance and hand motor speed. In MHE patients, high correlations were found between balance and FAST test, gait velocity and verbal skills, hand strength variability and anxiety and depression, and motor speed and FAST and TAVEC. MHE patients showed worse motor and cognitive performance than patients without MHE. MHE patients could have impaired movement control expressed as bradykinesia, and this reduced motor performance could correlate with cognitive performance.

## 1. Introduction

Liver cirrhosis is associated with multiple complications, the most serious of which is hepatic encephalopathy (HE) [1,2], a common reason for hospital admission [3]. Liver cirrhosis is also associated with frailty, leading to increased risk of sarcopenia, malnutrition, falls, fractures, hospitalization, limited recovery, low quality of life and death [4,5,6,7,8,9].

Overt HE is frequently preceded by minimal hepatic encephalopathy (MHE) [10,11,12,13,14,15], with attention deficits and mild cognitive impairment unveiled by psychometric tests [12,13,14,15]. Given that cognitive impairment and neuropathy in patients suffering from liver disease trigger onset of frailty, one line of research adopted involves frailty assessment using scales or psychometric tests. Ney et al. aimed to determine a specific cognitive and physical profile in these patients with the ultimate goal of identifying early markers of progressive deterioration [6]. These authors described a composite score from the Montreal Cognitive Assessment and the Clinical Frailty Scale to predict hospital admission of HE patients at six months [6]. However, motor evaluations like clinical tests or scales have certain limitations, as results can be biased by evaluator subjectivity or inaccurate patient reporting. In this regard, including the motor sign assessment in the study of early indicators in MHE patients is a step forward, insofar as biomechanical tools allow researchers to check objective measures of frailty. At the same time, these precise evaluations enable us to better prove the risk in non-disabled patients [4]. In this line, Mechtcheriakov et al. [16] analyzed the kinematics of handwriting in patients with liver cirrhosis and found that handwriting movement was markedly slower and less efficiently coordinated in patients than in healthy control subjects. Moreover, Urios et al. [8] found that cirrhotic patients with MHE showed impaired balance (mainly with eyes open on an unstable surface) compared to cirrhotic patients without MHE. Likewise, posturography test parameters were correlated with other biomechanical parameters like motor coordination and cognitive domains such as attention [8]. Although there seems to be motor differentiation between patients with and without MHE, to our best knowledge no studies have assessed motor performance with biomechanics tools, or which specific cognitive domains, related to motor disturbances, can discern between cirrhotic patients with and without MHE.

In light of the above, we hypothesize that cirrhotic patients with MHE have worse cognitive and physical performance than people with cirrhosis but without MHE. Furthermore, we believe that some cognitive outcomes could be correlated with biomechanical variables about the physical state of people with cirrhosis. To contrast the hypothesis raised, the main aim of this study was to identify cognitive and motor differences in patients with liver disease with and without MHE, using objective evaluation tools. As a secondary objective, we assessed potential correlations between motor signs and cognitive performance in both patient groups.

## 2. Materials and Methods

### 2.1. Study Design and Participants

The study was cross-sectional with a convenience sampling (specifically, modal instance sampling, i.e., volunteers with the pathology to study) design. Sample size was calculated considering biochemical variables (3-nitrotyrosine) that discriminate between cirrhotic patients with and without MHE, as well as resonance parameters obtained in previous studies and that presented less power of discrimination than 3-nitrotyrosine [17,18]. This analysis was performed using the STATA statistical program, comparing the means and standard deviations for each group and resulting in a number of 30 subjects for each group, with a significance level of 5% and a power of 80%.

Seventy-six patients with liver cirrhosis were consecutively recruited from the outpatient clinics of Hospital Clinico and Hospital Arnau de Vilanova from Valencia (Spain). The diagnosis of cirrhosis was based on clinical, biochemical and ultrasonographic data. Inclusion criteria were (i) chronic liver cirrhosis, (ii) able to stand and walk without use of assistive devices, (iii) stable medication and (iv) older than 18 years old. Exclusion criteria were (i) overt HE or history of overt HE, (ii) recent (<6 months) alcohol intake, (iii) history of abuse or drug-dependence of any another substances besides alcohol, (iv) history of disease or secondary trauma that may influence cognitive or motor deterioration, (v) chronic disease without normative treatment (visual problems, high blood pressure, diabetes, hypercholesterolemia). Ten patients did not want to collaborate or were unable due to scheduling reasons. Sixty-six participants completed this study.

All participants completed the Psychometric Hepatic Encephalopathy Score (PHES), a battery of five psychometric tests used for MHE diagnosis [14,15,19]. The PHES score was calculated, adjusting for age and educational level, using Spanish normality tables (www.redeh.org). Patients were classified as MHE when the score was ≤−4 points [15]. After performing the PHES, patients were stratified into 28 patients with MHE, and 38 patients without MHE. All participants were included after giving written informed consent. Study protocols were approved by the Scientific and Research Ethics Committees of Hospital Clinico Universitario and Arnau de Vilanova Hospital of Valencia, Spain (approval code: 2018/210; approval date: 2 March 2018) and were in accordance with the ethical guidelines of the Helsinki Declaration.

### 2.2. Procedures

The procedure was as follows: (1) recruitment of patients, collecting written informed consent and clinical interview to register participants’ main baseline characteristics, and collection of etiology data of liver disease and analytical values for the calculation of indicators of severity of liver disease (Child-Pugh score, model end-stage of liver disease, model end-stage liver disease (MELD) score); (2) classification of cirrhotic patients with the PHES score in patients without and with MHE, at the Digestive Medicine Unit of Hospital Clinico de Valencia; (3) anthropometric evaluation to register weight, height and length of lower limbs; (4) cognitive and psychological evaluation and (5) biomechanical assessment of gait, balance, hand strength and manual motor speed. All tests were carried out during a single evaluation session lasting between an hour and a half and two hours. Usually sixty minutes were earmarked for cognitive assessment and thirty minutes for biomechanical assessment. Tests were performed in the Unit of Personal Autonomy, Dependency and Mental Disorder Assessment at the Faculty of Medicine, University of Valencia (Spain).

### 2.3. Cognitive-Functional Assessment and Outcomes

The following tests were used to characterize cognitive and psychological performance:

1. Mini-Mental State Examination (MMSE), a series of tests to measure and grade cognitive impairment [20].
2. Verbal Fluency Test: consisting of two tasks [21], semantic verbal fluency [22,23] and phonemic verbal fluency [24], as well as a total score combining the two tests.
3. Aprendizaje Verbal España-Complutense Test (TAVEC): to measure learning and memory, consisting of different subtests: immediate memory with possibility of learning, immediate memory without possibility of learning, short-term free memory, short-term memory-guided or with semantic keys, long-term free memory, long-term memory-guided or with semantic keys, and long-term recognition [25,26]. In addition, an overall score could also be obtained by combining the short-term free memory and short-term memory-guided, termed global short-term memory, and combining long-term memory and long-term memory-guided, which is called global long-term memory.
4. Wechsler Adult Intelligence Scale III (WAIS): to evaluate intelligence by verbal memory and performance skills [25,27].
5. Hamilton Rating Scale: measures presence and severity of depression and anxiety: [28,29].
6. Functioning Assessment Short Test (FAST): clinically assesses psychological functioning in the following areas, (a) autonomy, (b) work, (c) cognitive, (d) finances, (e) relationships and (f) leisure [30]. It also calculates a total score of functioning.

### 2.4. Biomechanical Gait Assessment and Outcomes

We evaluated four functional aspects of motor performance related to deterioration in patients with liver disease [4,6,16,31]. The procedure followed in each one is defined below.

#### 2.4.1. Gait

Gait was measured with two photocells and two force platforms (Dinascan/IBV Biomechanical Institute of Valencia, Valencia, Spain) and NedAMH/IBV software (version 5.1.0, 2013, Biomechanical Institute of Valencia, Valencia, Spain). Participants walked barefoot along a 10 m-long corridor at a self-selected comfortable speed. The platforms were located at the center of the corridor to record the central step and avoid acceleration and deceleration at the start and end of the gait cycle. Gait outcomes were (1) gait velocity (m/s), (2) stance time (s), (3) braking force: minimum registered anterior–posterior ground reaction force (GRF) corresponding to heel strike (N), (4) propulsive force: maximum registered anterior–posterior GRF corresponding to take-off (N), (5) swing force: minimum registered vertical GRF occurring during contralateral foot oscillation (N), (6) push-off force: second highest register of vertical component of GRF that occurs between heel-off and toe-off (N).

#### 2.4.2. Balance

Functional postural balance was assessed using a single force platform (Dinascan/IBV Biomechanical Institute of Valencia, Valencia, Spain) and NedSVE^®^/IBV software (version 5.1.0, 2013, Biomechanical Institute of Valencia, Valencia, Spain) [8]. The Ned/SVE system evaluates center of pressure (COP) displacement during the Romberg tests for 30 s under four different stability conditions (eyes open or closed on a firm surface or with 10 cm-thick foam mattress under the feet) [32]. During the test, participants maintained bipedal posture with feet together, slightly open at a 45° angle without footwear, as shown in Supplementary Figure S1. Markings were used as a guide to ensure standardized foot positioning. We studied outcomes of the Romberg test with the most unstable condition (eyes closed with feet on a foam mattress): (1) COP displacement angle: displacement vector orientation (°) extending from the starting point to the end position of the subject; (2) total COP displacement: total distance of the COP reached from the origin (mm); (3) anterior–posterior COP displacement: maximum anterior–posterior distance reached by COP during the test (mm); (4) mediolateral COP displacement: maximum mediolateral distance reached by COP during the test (mm) and (5) COP swept area: area of subject’s swing, calculated from beginning until end of the test (mm^2^) [32].

#### 2.4.3. Hand Strength

Grip strength was measured with an electronic dynamometer (NedVEP/IBV Biomechanical Institute of Valencia, Valencia, Spain) and NedDiscapacidad/IBV software (version 4.1.1, Biomechanical Institute of Valencia, Valencia, Spain) [33,34,35]. The ergonomic position to perform the test was sitting upright in a chair with backrest but without armrests. Feet were supported on the floor with 90° knee flexion. The arm was positioned with 90° elbow flexion and neutral forearm pronosupination [36]. Hand strength was recorded for three functional positions during 30 s sustained force: (a) handgrip, (b) lateral pinch (thumb pad and side of index finger), (c) tip pinch (thumb and index finger) [33], as shown in Supplementary Figure S2. Three maximum strength scores from both left and right hands were obtained for each functional position. The repetitions in each hand did not differ by more than 10% and the average was calculated for each side [37]. The hand assessment outcomes were (1) grip strength: average of grip strength repetitions, for right and left hand individually, (2) lateral pinch strength: average of lateral pinch strength repetitions, for right and left hand individually, (3) tip pinch strength: average of tip pinch strength repetitions, for right and left hand individually, (4) coefficient of variation percentage (CV): standard deviation ratio to mean hand strength during each task, which indicated variability in hand force strength performance and strength stability control [38], (5) Index of Difference (ID): difference between the performance of the right hand minus the left hand.

#### 2.4.4. Manual Motor Speed

The Finger Tapping Test (FTT) was used to measure motor response speed. Each subject was required to tap a key as many times as possible using the index finger of each hand, and the number of taps was recorded [39]. Participants completed one test with the right hand, another with the left hand and then performed the test with both hands at the same time. The outcomes extracted from the test were (1) FTT right: number of times the key is pressed with the right hand, (2) FTT left: number of times the key is pressed with the left hand, (3) FTT_2_ right: number of times the key is pressed with the right hand while the test is performed with both hands, and (4) FTT_2_ left: number of times the key is pressed with the left hand while the test is performed with both hands.

### 2.5. Statistical Analysis

Statistical analyses were performed using IBM SPSS v.24 (SPSS Inc., Chicago, IL, USA). Standard statistical methods were used to obtain the mean and standard deviation (SD). A one-way between-subject factor multivariate analysis of variance was conducted to analyze the between-subject group factor effect with two categories (i.e., patients with and without MHE) on the dependent outcomes and the quantitative demographic variables. Ground reaction force variables were standardized according to anthropometric data, specifically, participants’ weight. Total scores of psychological and cognitive tests as well as the subscores of the domains or constructs that made up each scale were considered as different dependent variables, as described in Section 2.3. When significant factor effects were found, the Bonferroni correction was used for post-hoc (pairwise) mean comparisons. Differences were considered statistically significant if *p* < 0.05. On the significant results of the comparison between groups with and without MHE, a one-way between-subject factor multivariate analysis of variance was repeated, comparing the participants who had alcohol as the etiology of cirrhosis with those who had other etiologies. The relationships between cognitive and motor measures were explored using Pearson’s correlation tests (r) with a level of significance of *p* = 0.05. Correlation analyses were performed for each group studied. When significant correlations of the variables were found in the MHE group, the same pattern was checked in the group without MHE, and was statistically compared. As a further step, a chi-square test was performed to demonstrate between-group differences in gender, etiology of cirrhosis, ascites and esophageal varices.

## 3. Results

### 3.1. Participants’ Characteristics

Seventy-six patients were approached and performed the PHES battery. Forty-four were without MHE and 32 with MHE. Finally, sixty-six participants (38 without and 28 with MHE) completed this study. Patient characteristics are shown in Table 1. No significant between-group differences were observed in age, weight, height, body mass index or gender (*p* > 0.05). There were no significant differences in severity of liver disease, evaluated by Child–Pugh and MELD scores (*p* > 0.05). No patients had opioid or benzodiazepine medication. No significant differences were found in the comparison of the etiologies of cirrhosis (*p* = 0.052).

### 3.2. Cognition and Mental State

A significant effect of the group factor was observed on cognitive variables evaluated (*p* < 0.05), which is shown in Table 2.

In the FAST test, we only saw differences in the domains of autonomy and cognition (*p* < 0.05), not in other domains or in the total score (*p* > 0.05). Likewise, the MHE group performed significantly worse on the cognitive state (MMSE) (*p* < 0.05) and intelligence outcomes (WAIS test) (*p* < 0.05), indicating a lower cognitive ability performance. The MHE group showed significantly worse scores than patients without MHE in immediate memory and learning, short-term memory and long-term recognition. Finally, in the Verbal Fluency Tests, patients with MHE performed worse than the patients without MHE (*p* < 0.05) in both the semantic and phonemic subsections (Table 2).

### 3.3. Motor Performance

**Figure 1:**
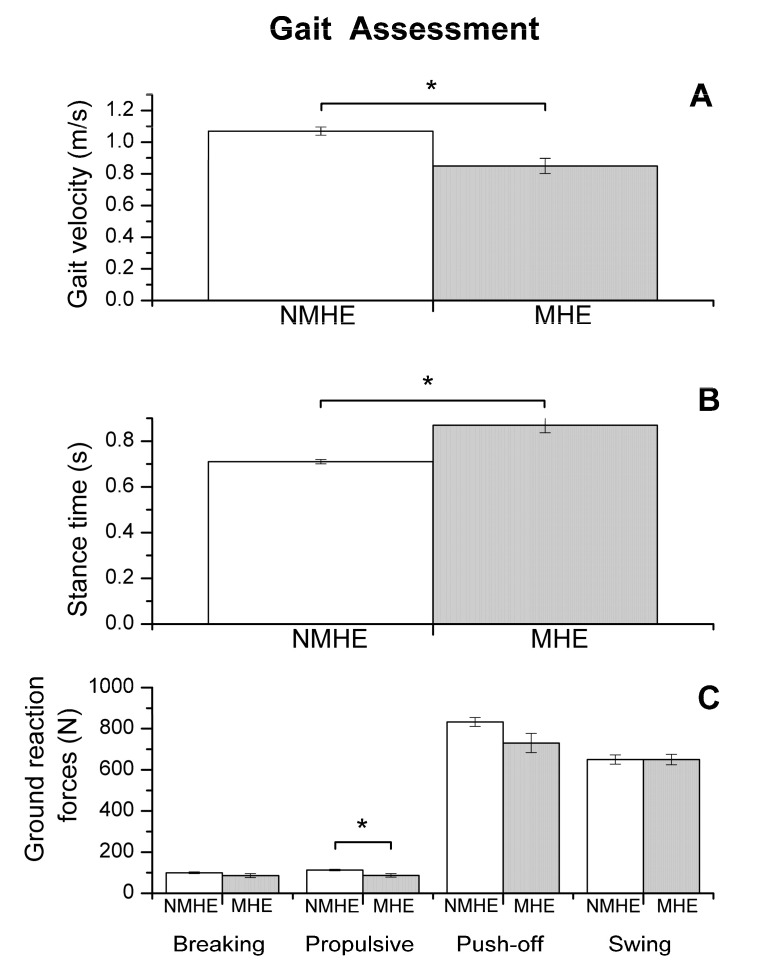
Biomechanics results: gait assessment (mean and standard error) in patients with minimal hepatic encephalopathy (MHE) and without MHE (NMHE). (**A**) Gait velocity; (**B**) stance time; (**C**) ground reaction forces: breaking, propulsive, push-off and swing. Asterisks indicate statistically significant differences between groups: * *p* < 0.05. Ground reaction force outcomes are shown without normalizing to subject weight.

**Figure 2:**
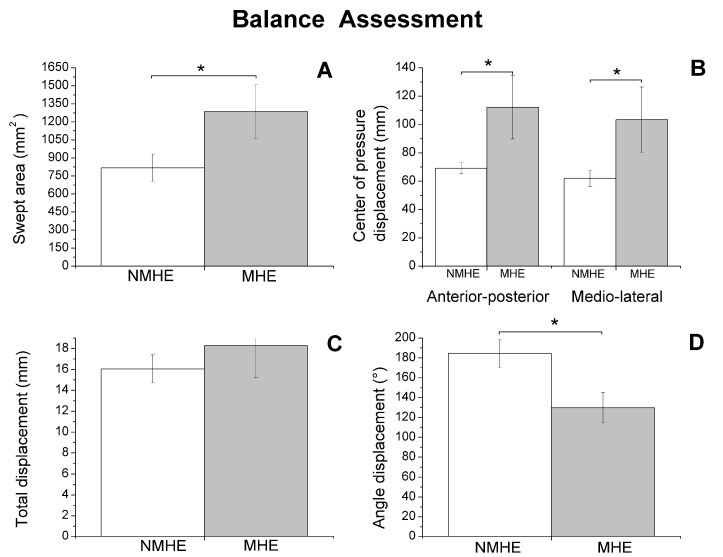
Biomechanics results: balance assessment (mean and standard error) in patients with minimal hepatic encephalopathy (MHE) and without MHE (NMHE) during Romberg test with a 10 cm foam under the feet, and eyes closed. (**A**) Swept area; (**B**) center of pressure displacement; (**C**) total displacement; (**D**) angle displacement. Asterisks indicate statistically significant differences between groups: * *p* < 0.05.

**Figure 3:**
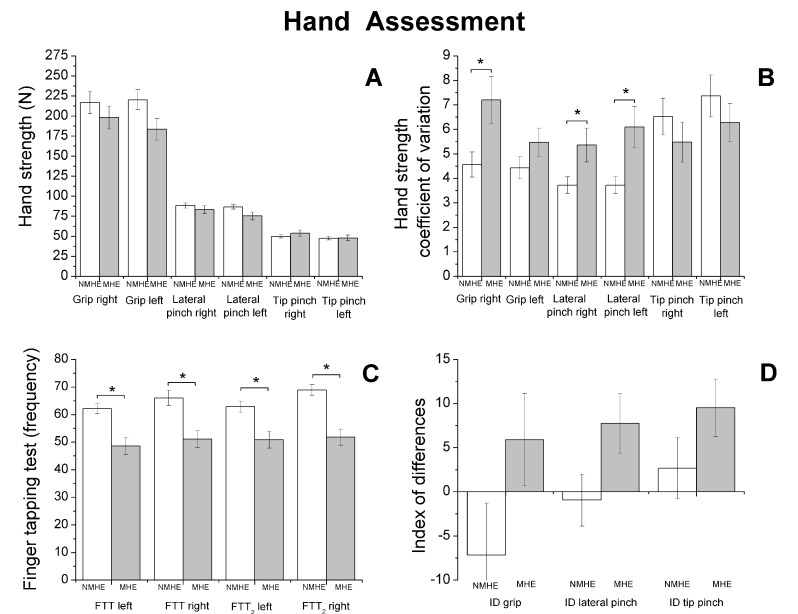
Biomechanics results. Hand strength performance (mean and standard error) in patients with minimal hepatic encephalopathy (MHE) and without MHE (NMHE). (**A**) Hand strength: grip, lateral pinch and tip pinch were measured bilaterally; (**B**) hand strength: coefficient of variation; (**C**) Finger Tapping Test (FTT) (frequency); (**D**) Index of Difference (ID) for hand strength. Asterisks indicate statistically significant differences between groups: * *p* < 0.05.

A significant group factor effect was also observed in the motor variables evaluated (*p* < 0.05), which is shown in Figure 1, Figure 2 and Figure 3.

Regarding biomechanics outcomes, we observed that MHE patients had slower gait velocity and showed reduced propulsion and take-off force (*p* < 0.05) at the end of the stance phase of the walking cycle (Figure 1). The MHE group also had worse balance in the standing position with visual and proprioceptive disturbance (Figure 2), with a significantly greater swept area and center of pressure displacement than patients without MHE (*p* < 0.05) (Figure 2A,B), and lower angle displacement (*p* < 0.05) (Figure 2D). Even though no differences were observed in hand strength in the different functional tests (*p* > 0.05) (Figure 3A), we noticed that the MHE group showed a greater variability on right hand grasping tests (*p* < 0.05) and in the test of lateral pinch with both hands (*p* < 0.05) (Figure 3B,D). Furthermore, MHE group participants showed a slower performance in the manual motor speed assessment of the Finger Tapping Test than patients without MHE (*p* < 0.05) (Figure 3C).

### 3.4. Correlation between Motor and Cognitive/Mental Performance

Table 3 shows the principal statistically significant correlations (*p* < 0.05) found in the analysis patients with and without MHE. The most significant correlations for the MHE group were between COP displacement and FAST test, specifically within the “work functioning” subsection (r = −0.81; *p* < 0.001) and with the total functioning score (r = −0.74; *p* = 0.01). Furthermore, in the MHE group, we observed an association between gait velocity and tests related to verbal skills, right lateral pinch coefficient of variation with anxiety and depression, right hand motor speed (bilateral performance) with the financial functioning of FAST test and last, left hand motor speed (bilateral performance) with the short-term memory and total score of the memory and learning test (Table 3). In the non-MHE patient group, associations were different; only gait velocity was significantly correlated with verbal fluency (*p* < 0.05).

Given that alcoholic etiology of cirrhosis could be a possible confounder and that the effect of alcohol abuse on the equilibrium even after a long abstinence is already well-known, it seems reasonable to assume that past alcohol abuse also plays a role in balance impairment and brain alterations. However, when we grouped the patients in the study according to etiology, there were no significant differences between cirrhotic patients of alcoholic etiology and other etiologies in the analyzed parameters of gait, balance, variability of hand strength or motor speed of the hand (*p* > 0.05), as shown in the Table S1 of Supplementary Materials.

## 4. Discussion

**Figure 4:**
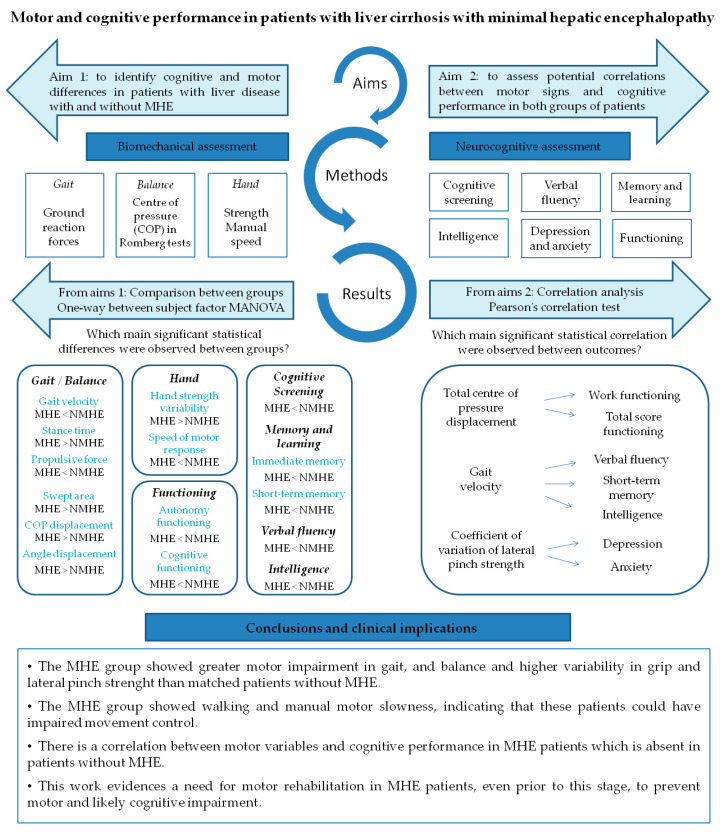
Conceptual model figure integrating the findings of this study.

This study brings to light three important findings. Figure 4 summarizes the findings of this study. Firstly, MHE patients showed worse performance in gait and balance when compared to patients without MHE, meaning worse balance during the unstable condition Romberg test, slower walking, a longer support phase of gait cycle and lower ground reaction force before swing phase. This directly affects the kinematics of the lower limb during the oscillation phase, since leg elevation from the floor will depend more on the patient’s muscular effort than on the mechanical impulse of propulsion and take-off force, leading to a higher risk of falls. Although differences were not found between groups’ hand strength performance, the MHE group showed a greater variability in gripping force performed with the right hand and on the lateral pinch with both hands. These results agree with Lauridsen et al., who demonstrated variability in motor performance, by the greater variability of motor reaction times in MHE patients [40]. In addition, concurrent with previous findings [16,18], MHE patients underperformed in all the manual motor speed tests evaluated when compared with patients without MHE. Although we cannot assert that MHE subjects from our study are fragile [41], we observe that they suffered from a lack of motor control, as evidenced by bradykinesia and variability of movement during physical tests. Bradykinesia is characteristic in a spectrum of neurological diseases such as Parkinson’s disease, but is also a hallmark of normal aging. Nonetheless, slowness associated with impaired accuracy more likely represents a pathological condition [42], such as MHE, where ammonia and other endogenous substances (resulting from an impaired hepatic metabolism) affect brain structure and functionality [43], causing a bradykinetic pattern like in Parkinson’s disease [44]. These findings support the results of other studies related to the slowness and inaccuracy demonstrated through the Line Tracing Test and Continuous Reaction Time Test [40,45], and raises the alteration of motor control in patients with MHE prior to physical frailty.

The second important finding was that in the MHE group of patients, motor impairment goes hand-in-hand with the worst of all cognitive performance (Figure 4). We observed a poorer performance in the MHE group than in patients without MHE in functioning, memory, verbal semantic and phonetic fluency and general cognitive tests, such as the MMSE. This was expected given that the MHE classification itself is based on a cognitive battery (PHES score) [15]. However, this study conducts a more in-depth analysis of the most frequently impaired cognitive domains through the most commonly cognitive state characterization tests. For example, in functionality as evaluated by the Functioning Assessment Short Test, we noticed that patients differ specifically in the domain of autonomy, suggesting that the MHE group is less capable than patients without MHE of performing housework, living alone, buying food or taking care of themselves. These results agree with previous studies showing an impaired daily functioning in patients with MHE which would negatively affect the quality of life of MHE patients [9,46]. Conversely, these functional differences were not observed in interpersonal relationships or leisure domains.

Finally, some motor variables were found to be intimately correlated with cognitive aspects and psychological functioning, in which the variables with the highest proportion of variance share (r^2^) were COP displacement and functioning test, specifically with the “work functioning” subsection (r = 0.66) and with the total score (r = 0.55) (Figure 4). This finding is especially interesting as it clarifies the specific areas of functioning where MHE patients may have greatest difficulties. In this regard, Åberg et al. [47] reported that having gainful employment is one of the major factors influencing perceived quality of life in patients with liver disease. Another study has established similar correlations but using physical clinical tests such as the Chair Stand Test and Unipedal Stance Time Test, which were correlated with cognitive tests of executive function [48].

We found that gait speed is associated with performance on verbal fluency and memory tests in patients with MHE. Functional ambulation requires coordinated motor and cognitive skills. Rao et al. reported high levels of interference between gait and cognition in people with essential tremor [49]. Brain imaging studies showed that gait speed and performance on executive functions and memory tests are dependent on prefrontal cortex activation [50,51], where functional connectivity is decreased in MHE patients, leading to cognitive deficits [52].

In different studies, motor deterioration in patients with liver disease has been established and signaled as a predictor of worse patient outcomes. For example, Lai et al. [53] found that frail cirrhotic patients had poorer performance in gait, strength and functionality, and proposed a new frailty index from grip strength, chair stands and balance that could predict which cirrhotic patients listed for liver transplantation would die or be delisted from transplant due to illness [54]. In this regard, it is plausible that biomechanical variables could serve as MHE biomarkers or at least as early indicators of pre-MHE stages. These areas need clarification and warrant future studies.

This work evidences a need for motor rehabilitation in MHE patients, even prior to this stage, to help prevent motor deterioration. Further, we postulate that physiotherapy to improve balance, motor speed and facilitation of gait oscillation, may also simultaneously improve cognitive domains such as verbal memory and overall cognitive performance. Although research has shown that cognitive training improves not only cognitive abilities but also motor skills in patients with neurodegenerative diseases [55], the correlations found in this study provide new evidence that the reverse may also be true, and motor training may improve cognitive abilities.

## 5. Conclusions

In this study, we demonstrate that MHE patients show greater motor impairment in gait and balance, and present higher variability in grip and lateral pinch strength than age, gender, height and weight-comparable liver disease patients without MHE. The MHE group showed walking and motor manual slowness, indicating that these patients could have impaired movement control expressed as bradykinesia. We also found a significant correlation between motor variables and cognitive performance in MHE patients, which was absent in the non-MHE group.

## Figures and Tables

**Table 1:** Clinical and demographical variables of participants.

Demographical and Clinical Variables	Patients with MHE (*n* = 28)	Patients without MHE (*n* = 38)	Between Groups
Age (years) ^a^	63.42 (9.17)	61.12 (8.34)	0.52
Weight (kg) ^a^	79.56 (13.35)	79.36 (13.14)	0.83
Height (m) ^a^	1.66 (0.07)	1.66 (0.07)	0.98
BMI ^a^	28.68 (3.90)	28.82 (4.81)	0.75
Child–Pugh score (A/B/C) ^b^	20/7/1	28/9/1	0.76
MELD score ^a^	9.7 (2.6)	9.4 (3)	0.66
Gender	M:21/F:7	M:30/F:8	0.70
Etiology of cirrhosis			0.05
Alcohol ^b^	14	20	
HCV/HBV/HCV + alcohol ^b^	4/1/1	13/0/1	
NASH ^b^	5	4	
Primary biliary cirrhosis ^b^	2	0	
Autoimmune	1	0	
Ascites ^b^	12	8	0.08
Esophageal varices ^b^	19	18	0.20

^a^ Values are expressed as mean (SD). ^b^ Values are expressed as frequency. MHE, minimal hepatic encephalopathy; BMI, body mass index; MELD, model end-stage liver disease; Gender, male (M) and female (F); HCV, hepatitis C virus; HBV, hepatitis B virus; NASH, non-alcoholic steatohepatitis.

**Table 2:** Descriptive and inferential statistical analysis results of cognitive variables evaluated.

Outcomes	Patients with MHE		Patients without MHE		Post-Hoc *p*-Value
	Mean	SD	Mean	SD	
Functioning Assessment Short Test (FAST)					
Autonomy functioning	4.43	3.17	2.74	1.98	0.03 *
Work functioning	13.18	4.78	12.76	4.90	0.16
Cognitive functioning	6.14	3.96	3.16	2.11	0.00 *
Finances functioning	1.14	1.63	0.55	1.03	0.40
Relationships functioning	4.93	3.87	5.13	2.88	0.35
Leisure functioning	3.00	2.34	1.89	1.81	0.10
Total functioning	32.82	12.80	26.24	9.77	0.19
Hamilton depression and anxiety rating scale					
Depression	6.71	6.33	6.71	4.77	0.76
Anxiety	8.86	9.66	8.32	5.23	0.83
Mini-Mental State Examination Test (MMSE)					
Cognitive state	26.35	2.88	28.13	1.42	0.03 *
Aprendizaje Verbal España-Complutense Test (TAVEC)					
Immediate memory and learning	34.26	9.65	39.39	9.85	0.02 *
Immediate memory	3.19	1.27	3.95	1.39	0.02 *
Short-term memory	5.52	3.27	8.26	2.70	0.01 *
Short-term memory-guided	7.26	2.89	9.24	2.82	0.09
Global short-term memory	12.78	5.32	17.24	5.30	0.05
Long-term memory	6.78	2.91	8.53	2.81	0.05
Long-term memory-guided	7.56	2.89	9.16	3.03	0.14
Global long-term memory	14.33	5.28	17.53	5.60	0.10
Long-term recognition	13	2.44	13.61	1.94	0.04 *
Verbal Fluency Test					
Semantic verbal fluency	13.96	4.04	20.38	5.46	0.00 *
Phonemic verbal fluency	19.93	9.41	33.81	10.94	0.00 *
Total verbal fluency	33.89	12.30	54.19	14.43	0.00 *
Wechsler Adult Intelligence Scale III (WAIS)					
Intelligence	33.19	12.39	43.37	7.17	0.00 *

The table shows mean SD value for the variables measured with psychometric instruments on cognition and functionality for both groups of the study. In addition, the *p*-value of the multivariate analysis of variance is shown to observe the differences between groups. * Indicates statistically significant differences.

**Table 3:** Pearson correlation for cognitive outcomes and motor performance.

Cognitive Outcomes	Patients with MHE	Patients without MHE
	Gait Velocity	
Short-term memory (TAVEC test)	0.51 (0.02) *	0.27 (0.11)
Semantic verbal fluency (Verbal Fluency Test)	0.66 (0.00) *	0.41 (0.01) *
Phonemic verbal fluency (Verbal Fluency Test)	0.61 (0.00) *	0.41 (0.01) *
Intelligence (WAIS test)	0.50 (0.05) *	0.03 (0.87)
Total center of pressure displacement		
Work functioning (FAST test)	−0.81 (0.00) *	−0.12 (0.49)
Total functioning (FAST test)	−0.74 (0.01) *	0.07 (0.69)
Right lateral pinch coefficient of variation		
Depression (Hamilton test)	0.54 (0.01) *	0.27 (0.11)
Anxiety (Hamilton test)	0.52 (0.02) *	0.21 (0.22)
Right hand motor speed (bilateral performance)		
Finances functioning (FAST test)	0.50 (0.04) *	0.31 (0.06)
Left hand motor speed (bilateral performance)		
Short-term memory (TAVEC test)	0.53 (0.02) *	0.12 (0.49)
Total score of memory and learning (TAVEC test)	0.48 (0.04) *	0.21 (0.23)

This table shows the results of the correlation analysis between motor and cognitive variables. Values in the table indicate Pearson value (*p*-value). * Indicates statistically significant correlations (*p* < 0.05). FAST, Functioning Assessment Short Test; HD, Hamilton Depression Rating Scale; HA, Hamilton Anxiety Rating Scale; MHE, minimal hepatic encephalopathy; TAVEC, Aprendizaje Verbal España-Complutense Test; VFS, Verbal Fluency Semantic; VFP, Verbal Fluency Phonemic; WAIS, Wechsler Adult Intelligence Scale III.

## Supplementary Materials

The following are available online at https://www.mdpi.com/2077-0383/9/7/2154/s1, Figure S1: Balance assessment; Figure S2: Grip strength; Table S1: Pairwise comparison between participants with alcoholic etiology of cirrhosis and participants with other diseases.

## Abbreviations

COP	center of pressure
CV	coefficient of variation percentage
FAST	Functioning Assessment Short Test
FTT	Finger Tapping Test
GRF	Ground reaction force
HA	Hamilton Anxiety Rating Scale
HD	Hamilton Depression Rating Scale
HE	hepatic encephalopathy
ID	Index of Difference
MHE	minimal hepatic encephalopathy
MMSE	Mini-Mental State Examination
PHES	Psychometric Hepatic Encephalopathy Score
TAVEC	Aprendizaje Verbal España-Complutense Test
VFP	Verbal Fluency Phonemic
VFS	Verbal Fluency Semantic
WAIS	Wechsler Adult Intelligence Scale III
