# Supplementary material for: Motor and Cognitive Performance in Patients with Liver Cirrhosis with Minimal Hepatic Encephalopathy
Source: J Clin Med. 2020 Jul 8;9(7):2154. doi: 10.3390/jcm9072154 (PMC7408738; doi:10.3390/jcm9072154)
Supplement: Supplementary file 1 [file jcm-09-02154-s001.pdf]

SUPPLEMENTARY MATERIAL

Motor and cognitive performance in patients with liver cirrhosis with minimal hepatic encephalopathy

Constanza San Martín-Valenzuela, Aroa Borras-Barrachina, Juan-José Gallego, Amparo Urios, Víctor Mestre-Salvador, Patricia Correa-Ghisays, María-Pilar Ballester, Desamparados Escudero-García, Joan Tosca, Cristina Montón, María-Pilar Ríos, Elena Kosenko, Vicente Felipo, Rafael Tabares-Seisdedos, Gabriel Selva-Vera* and Carmina Montoliu*

Contents:

- **Figure S1.** Balance assessment
- **Figure S2.** Grip strength
- **Table S1.** Pairwise comparison between participants with alcoholic etiology of cirrhosis and participants with other diseases

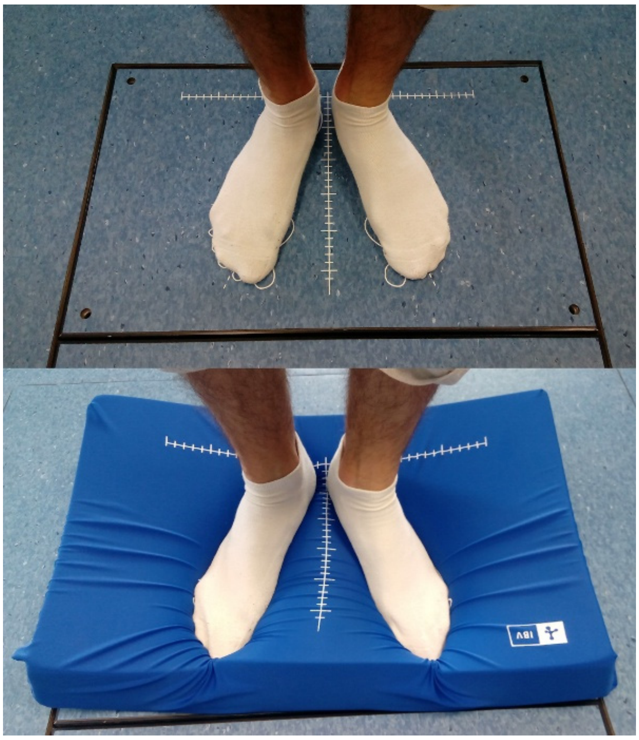

Figure S1. Balance assessment. A dynamometric platform is shown, with markings to ensure a standardized test (subject in bipedal position with feet together slightly open at a 45° angle without footwear).Four Romberg trials were measured under different stability conditions (eyes open or closed on a firm surface or with a 10 cm foam under the feet).

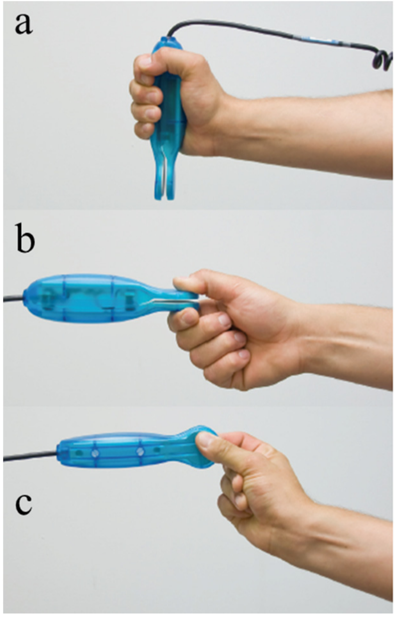

Figure S2. Grip strength. The hand strength was recorded for three functional positions during 30 s of force maintenance: a) handgrip, b) lateral pinch (thumb pad and lateral aspect of index finger), c) tip-pinch (thumb and index fingers).

Table S1. Pairwise comparison between participants with alcoholic etiology of cirrhosis and participants with other diseases

**Outcomes**	**(I) Etiology**	**(J) Etiology**	**Mean differences**	**Typical error**	**Statistical sig. (*p*-value)[a]**	**95% confidence interval for the difference [a]**	
Gait velocity	Alcohol	Other	,048	,057	,407	-,068	,164
Stance time	Alcohol	Other	-,002	,041	,964	-,084	,081
Propulsive ground reaction force	Alcohol	Other	13,003	9,882	,196	-6,969	32,976
Swept area	Alcohol	Other	13,248	261,917	,960	-516,107	542,602
Medio-lateral centre of pressure displacement	Alcohol	Other	8,471	21,812	,700	-35,613	52,555
Antero-posterior centre of pressure displacement	Alcohol	Other	4,001	20,425	,846	-37,279	45,281
Angle displacement	Alcohol	Other	-6,831	25,620	,791	-58,612	44,950
Hand strength coefficient of variation, grip right	Alcohol	Other	-,084	1,156	,942	-2,421	2,253
Hand strength coefficient of variation, grip left	Alcohol	Other	-,179	,898	,843	-1,993	1,635
Hand strength coefficient of variation, right lateral pinch	Alcohol	Other	,497	,958	,607	-1,439	2,433
Hand strength coefficient of variation, left lateral pinch	Alcohol	Other	-,600	1,125	,597	-2,873	1,673
Hand strength coefficient of variation, right tip pinch	Alcohol	Other	,505	1,330	,706	-2,183	3,193
Hand strength coefficient of variation, left tip pinch	Alcohol	Other	-,946	1,358	,490	-3,690	1,798
Finger tapping test, left	Alcohol	Other	-6,233	4,276	,153	-14,875	2,409
Finger tapping test, right	Alcohol	Other	-3,901	5,094	,448	-14,196	6,394
Finger tapping test 2, left	Alcohol	Other	-1,499	4,547	,743	-10,689	7,691
Finger tapping test 2, right	Alcohol	Other	-2,607	3,799	,497	-10,286	5,072

The table indicates the pairwise comparison of the one-way between-subject factor multivariate analysis of variance when participants are regrouped between those who have alcohol as the etiology of cirrhosis (alcohol) and those who have other pathology as the etiology of cirrhosis (Other). [a]Adjustment for multiple comparisons: Bonferroni.
